# Supplementary material for: Identification of the Core Promoter and Variants Regulating Chicken CCKAR Expression
Source: Genes (Basel). 2022 Jun 18;13(6):1083. doi: 10.3390/genes13061083 (PMC9222909; doi:10.3390/genes13061083)
Supplement: Supplementary file 1 [file genes-13-01083-s001.zip › genes-1766099-supplementary.pdf]

Table S1 Primers used to resequencing of 21 kb region around the *CCKAR*

Name	Position on chromosome 4 [1]	Forward primer (5'-3')	Reverse primer (5'-3')
CCKAR_up5k_O	73195458-73196287	CCCAAACATCCAACTTCCAA	TTTTGTTGTTTGACTCTTGCTCTT
CCKAR_up5k_N	73196237-73197039	AAAAGGGCCTGGAGATTATCA	CATTGTGTTGAGGGACATGG
CCKAR_up5k_M	73196971-73197755	CCATTCCAACTCTCCAAGCA	GTGAGAAGAGACCAACCCCA
CCKAR_up5k_K	73197549-73198252	AGTACAGAAAGGACATTGAGGTG	GGTCTTCCTCTGGATCTGCT
CCKAR_up5k_B	73198149-73198848	TCTGCTCTGCCCTTGTCAG	GGGTTTGTTGCCAGATCTTTC
CCKAR_up5k_C	73198698-73199363	TCCACAGAATCAATGGCCTT	GGGATGTTGGGAATTTTAGGCA
CCKAR_up5k_D	73199217-73199906	ACATTTCTCTAGACTACCTGCAG	CTGGCTCTTCATCTCAAAGGT
CCKAR_up5k_E	73199766-73200417	AATCCAGCTCAGTCCAGGAC	ATAGCTGAGACAAGGCTTCC
CCKAR_up5k_F	73200254-73200889	TGCCAGAAAGAACCAGGAGA	CTTCCATGAGCACTGTGGC
CCKAR_up5k_L	73200644-73201569	AAGAGATTGCTGCAGTTACGA	TACTCTGACCTGCTGCAAAC
CCKAR_up5k_H	73201354-73201992	ACGAAGCTGAAAACACATCCA	ATCCCAAACGTCTGAGTGGC
CCKAR_up5k_I	73201830-73202452	AGTTTTGGCATCTTAGACTGGA	ACTTGCAAACAGGATGTGCA
CCKARupstream	73202367-73203453	TACCCTTGAGGCTGGAAATG	ACCCTCTTCTGTTACTGGCC
CCKAR_exon1	73203262-73203789	GTGGTGTTGGTTGAGAGACG	CAGCAAAGCAGTGATGTTGG
CCKAR_exon1b	73203691-73204318	AACCAGCCTTCTTCCTAGCAG	GTGCACTGCTCATTCACCAC
CCKAR_intron1	73203781-73204630	GCTTTGCTGTGTGATATCCTCT	ACAATACAGCAGGATCCGGA
CCKAR_exon2	73204497-73204903	GGCTTTTGAAAGGGTGTACCT	TTCTCACATACCCCACTGGTT
CCKAR_intron2D	73204746-73205734	TAATGCTCTGCCTCTTCTGC	GCTGAAATGTGCAGAATCGG
CCKAR_intron2B	73205578-73206420	GAACCAGTACAAATAAAGGCTGT	TGAAAGCAGAAGGAAGGCAC
CCKAR_intron2C	73206231-73207058	CACCAATCAAGCAGGACAAGT	AGCGTACATGTTTCCAGATGG
CCKAR_exon3	73206618-73207199	CATTTGAAAACAGCAGAAGCA	TTACAGCAGGGGCTTAGCAG
CCKAR_intron3C	73206857-73207748	GCAAGCTGGTCCCTTTCAC	ACCTGTATCCTTCCTCCTCAC
CCKAR_intron3B	73207573-73208420	TGCTTCAACTGGTGCTGAGA	GCCTCAAGATGACTGCTCAC
CCKAR_exon4	73207950-73208628	AGGCACTCCCCTTTAAGAGC	GGTCCTAATCCACAGCCAGA
CCKAR_intron4	73208327-73209281	TTGATACCAGCCAGAGAAGATC	GTGTAGGACAGCAGGTGGAT
CCKAR_exon5	73208763-73209391	AATACTGGTGGCCAGCAAAC	CTCTAATCACAGGCGGCTTC
CCKAR_exon5b	73209117-73209622	GGCCAAGAAACTTGTCATCC	CTCACAGCGTTTACTGTCAGC
CCKAR_3'UTR	73209326-73210348	GTTTCCGCATGGGTTTTCTA	TTCAGCCTTATCCCTGTGCT
CCKAR_down5k_A	73210260-73211236	CACCCATGCATGTAAAGGGC	GTCTCATCCTGCAGCCTGA
CCKAR_down5k_B	73211174-73212124	ACGGGATTTAGTTCGTAACAGTG	GTTACCAACCTGTTCTGCTCA
CCKAR_down5k_C	73212061-73213031	TCAGATACTGCTCTCGATGGA	AGGCTGCCTTGGATATCTACC
CCKAR_down5k_D	73212892-73213891	ATCAGCAGCCTCCACATCAT	TTCTGGGTAGTCTCGTGTGG
CCKAR_down5k_E	73213797-73214742	CAGGCAGAACGTAGCATTGT	TGCATTCAAAAGGGAAGGGA
CCKAR_down5k_F	73214649-73215637	AAAACAATGCAGTCCAGGGG	TTAAAACCATGCTGCTCCGG
CCKAR_down5k_H	73215507-73216805	TTCACCAACAGCCCACTAC	TGTATGAGGGAAGCTGCGC

[1] Position of fragments is shown based on the galGal6.0 chicken genome assembly.

Table S2 Information of variants within the 21 kb region around the CCKAR

order	Physical position on chromosome 4 [1]	Position relative to CCKAR	dbSNP Access No.	High-growth allele [2]	Low-growth allele [2]	Variants studied in reporter gene assay
1	73196337	5' flanking region	rs736847850	T	C	
2	73196383		rs317607465	G	T	
3	73196641		rs732495896	T	C	
4	73196708		rs16434830	C	G	
5	73196743		rs740521921	C	T	
6	73196745		rs730956454	T	C	
7	73196779-73196780		novel	TG	CA	
8	73196831		rs731668344	C	G	
9	73196845		rs741200827	C	T	
10	73196891		rs739070235	A	G	
11	73196961		rs16434831	G	A	
12	73196979		rs16434832	G	A	
13	73197038		rs735487023	T	C	
14	73197046		rs738864180	C	T	
15	73197067		rs739505529	A	G	
16	73197317		rs732507897	A	T	
17	73197353		rs735868663	G	C	
18	73197354-73197355		novel	CACTTGG	del	
19	73197356		novel	G	C	
20	73197767		rs741249832	C	T	
21	73197824		rs732324989	A	G	
22	73198024		rs14489886	T	C	
23	73198364		rs738332766	A	T	
24	73198367		rs741716927	T	G	
25	73198431		rs731434187	T	A	
26	73198450		rs738921885	A	G	
27	73198468		rs739517525	G	A	
28	73198532		rs739346556	C	T	
29	73198560		rs736628093	G	A	
30	73198969		rs735180511	A	G	
31	73199139		novel	A	del	
32	73199343		rs732342426	A	G	CSNP1
33	73199485		rs740461407	T	G	
34	73199573		rs14489888	G	A	CSNP2
35	73199919		rs734291116	G	A	
36	73199976		rs733458406	del	C	
37	73200031		rs741747714	T	del	

38	73200087		rs741769582	G	A	
39	73200156		rs731441446	T	C	
40	73200187		rs315346474	G	T	
41	73200408		rs731258025	C	T	
42	73200656		rs735879555	T	C	
43	73200759		rs740873708		AAA	
44	73200862		rs731781640	C	T	
45	73200869		rs736179655	G	A	
46	73201089		rs739862145	A	G	
47	73201818		rs317340954	C	T	PSNP1
48	73201849		rs312791200	A	G	PSNP2
49	73202211		rs80637605	G	A	PSNP3
50	73202255		rs80691628	A	G	PSNP4
51	73202352		rs735709526	T	C	PSNP5
52	73202359		rs80644257	C	G	PSNP6
53	73202606		rs312284610	C	A	PSNP7
54	73202645		rs316320621	C	G	PSNP8
55	73202678		rs314545738	A	T	PSNP9
56	73202789		rs315057890	G	A	PSNP10
57	73202903		rs313514148	T	A	PSNP11
58	73202945		rs317602219	T	A	PSNP12
59	73203073		rs315801080	AC	TG	PSNP13
60	73203080		rs313590990	C	G	PSNP14
61	73203170		rs794017713	G	A	PSNP15
62	73203188		rs80606852	G	A	PSNP16
63	73203705	5'UTR	rs313822901	A	C	PSNP17
64	73203819	Exon1	rs314084118	T	C	PSNP18
65	73204044	Intron1	rs315176011	C	T	PSNP19
66	73204457		rs733514605	T	G	
67	73204458		rs794546486	G	A	
68	73204544		rs313134890	A	C	
69	73204550		rs314414260	T	C	
70	73204977	Intron2	rs316160134	C	G	
71	73204990		rs314874785	C	G	
72	73205026		rs315156020	C	T	
73	73205061		rs315703810	T	C	
74	73205162		rs316781477	C	A	
75	73205164		rs316193938	T	A	
76	73205380		rs740298767	T	C	
77	73205652		rs317439365	A	G	
78	73205673		rs13774462	A	C	
79	73205750		rs13774463	C	T	CSNP3
80	73205766		rs15616835	A	G	
81	73205821		rs13774464	T	C	

82	73205867		rs13774465	T	C	
83	73206071		rs314128048	A	G	
84	73206185		rs733004022	A	T	
85	73206488		rs314082180	T	C	
86	73206650		rs314888442	G	A	
87	73206696	Exon3	rs315278615	C	T	
88	73206714		rs315131298	C	T	
89	73206958	Intron3	rs315744114	G	A	
90	73206972		rs315844714	G	A	
91	73207405		rs316313304	T	C	
92	73207592-73207605		g.73207592-73207605 GATGTAAGATTTTAA>AAGATGTAAGAT	AAGATGTAAGAT	GATGTAAGATTTTAA	
93	73207637		rs317244862	A	G	
94	73207665		rs317125667	T	C	
95	73207680		rs734524617	C	T	
96	73207685		rs736521747	T	C	
97	73207696		rs317828971	A	G	
98	73207763		rs318084470	A	C	
99	73207790		rs317922543	A	G	g.73207790 A>G
100	73208653	Intron4	rs14489895	T	C	
101	73208701		rs14489896	G	A	
102	73208771		rs316271214	T	C	
103	73208780		rs316578408	G	A	
104	73208826		rs313367589	del	T	
105	73208894		rs313682508	G	A	
106	73208913		rs316541372	G	A	
107	73209189	Exon5	rs317682933	C	T	
108	73210442		rs740051404	T	C	
109	73210665		rs317824030	A	G	
110	73211281		rs16434839	A	G	
111	73211321		rs13774469	T	C	
112	73211362		rs13774471	G	T	
113	73211482	3'	rs313642761	C	A	
114	73211972	downstream	rs316810719	T	C	
115	73212017	region	rs316133489	A	C	
116	73212039		rs316773975	G	A	
117	73212039		rs794449055	G	A	
118	73212050		rs741772450	C	A	
119	73212572		rs316576898	T	A	
120	73212588		rs317722924	A	G	

121	73212595	rs317608264	C	T	
122	73212671	rs318192845	G	A	
123	73212782	rs312286641	T	C	
124	73212799	rs312783383	C	A	
125	73212803	rs313438885	C	A	
126	73212862	novel	G	C	
127	73213423	rs315186808	T	G	
128	73213450	rs14489908	G	A	
129	73213479	rs14489909	C	T	
130	73213609	rs316100778	T	C	
131	73213614	rs317332365	G	A	
132	73213728	rs738691378	C	T	
133	73213730	rs16434842	T	C	
134	73213732	novel	A	C	
135	73213740	rs16434843	A	T	
136	73213756	novel	C	T	
137	73213758	novel	A	T	
138	73213822	novel	A	C	
139	73214036	rs736432740	T	C	
140	73214037	rs741144618	G	A	
141	73214104	rs314260446	C	T	
142	73214106	rs316589852	C	T	
143	73214540	rs731882782	T	C	
144	73214886	rs313925792	G	A	
145	73214983	rs13774475	T	C	
146	73215008	rs13774476	A	G	
147	73215026	rs13774477	G	A	
148	73215300	rs737890322	G	A	
149	73215333	rs731102496	T	C	
150	73215349	rs734486715	T	C	
151	73215352	rs731723853	C	T	
152	73215400	rs737837005	T	C	
153	73215404	rs739853481	A	T	
154	73215409	novel	TAA	CCT	
155	73215430	rs730883312	C	G	
156	73215448	rs739641522	G	A	
157	73215528	rs738315002	G	A	
158	73215540-73215541	novel	TCGTCGGCT	del	CSNP4
159	73215738	rs313971226	C	T	
160	73215877	rs314426816	G	A	CSNP5
161	73215889	rs14489910	T	C	
162	73215916	rs14489911	T	C	
163	73215980	rs80599842	A	G	

164	73216019	novel	del	GTTTT
165	73216035	rs14489913	A	G
166	73216077	rs14489914	A	G
167	73216106	rs14489915	A	G
168	73216117	rs14489916	G	A
169	73216147	rs313442202	G	A
170	73216150	rs80613272	C	T
171	73216246	rs80649019	G	C
172	73216279	rs736014693	AA	G
173	73216321	rs736601394	C	T
174	73216431-73216566	novel	del	136 bp sequence[3]
175	73216652	rs734398941	G	A
176	73216683	novel	AC	GT
177	73216697	novel	CT	GA
178	73216702	rs731612315	T	A
179	73216713	rs738375545	T	C
180	73216720	rs741746296	T	C
181	73216733	rs731451820	C	G
182	73216947	rs313418816	G	A

[1] Physical positions of variants are given based on the UCSC galGal6.0 genome assembly.

[2] The allele is present in the High-growth or Low-growth haplotypes, respectively.

[3] 136 b sequence =
ATCATAGAATCGCTAAGGTTGGAAAAGACCCACAGGATCATCCAGTCCAACCATCCGCCCTTCATCAATGGTTCTCGCT
AAACCATGTCCCTCAACACAACATCCAAACGCTCTTTAAACACTACCAGGCTCGGTG

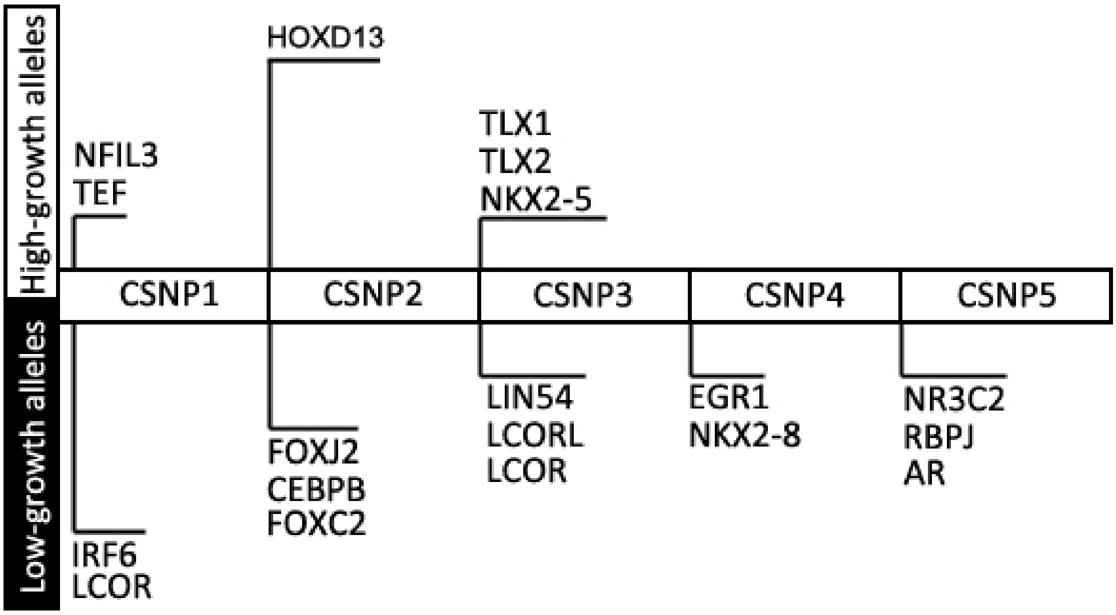

Figure S1 Effects of five conserved element variants on TF binding motifs

High- and low-growth alleles represent alleles contained in the high- and low-growth haplotypes, respectively. TFs at two sides of the bar represent that their motifs are created by high- or low-growth alleles.

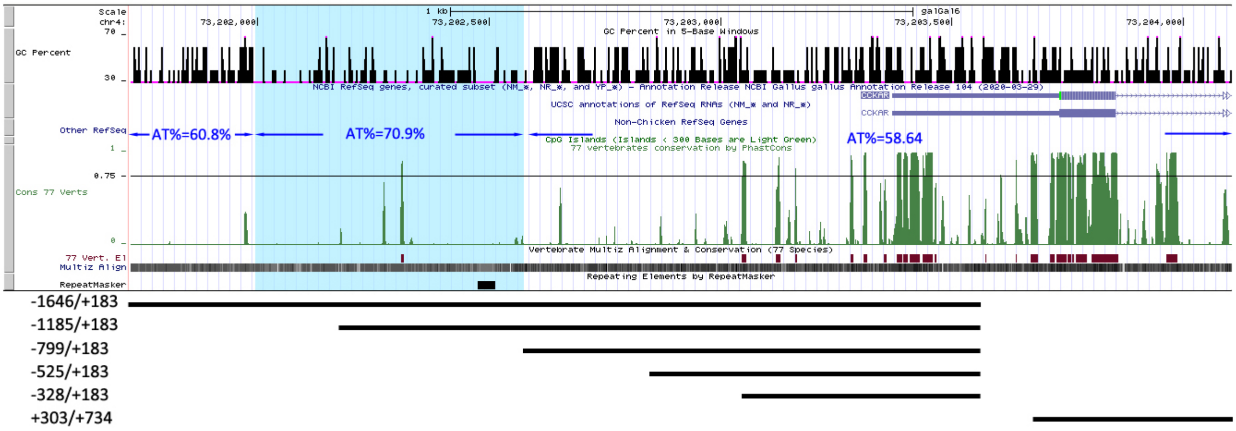

Figure S2 Distribution of GC content in upstream region of chicken *CCKAR* gene

The screen shot of UCSC Genome Browsers showed GC content and conservation tracts in -1646 bp~+734 bp from the TSS of *CCKAR*. An AT-rich region exists in this region and is highlighted in light blue. Beneath this plot, black lines represent fragments analyzed in the reporter assay. At the left of black lines the numbers indicate the nucleotide positions of the 5′-end/3′-end of fragments relative to the TSS.

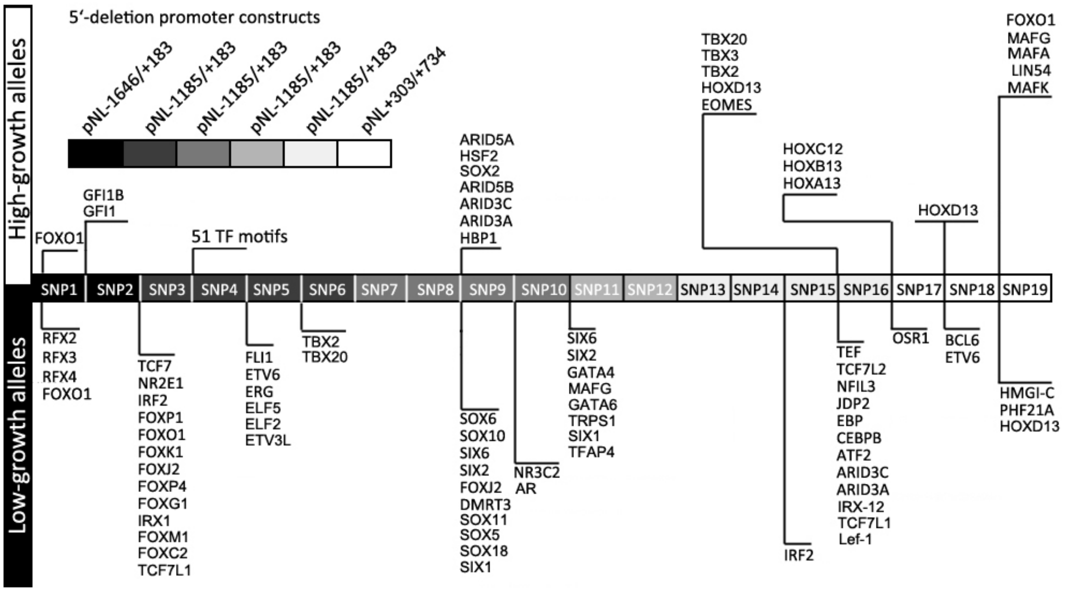

Figure S3 Effects of promoter variants of *CCKAR* on TF binding motifs

High- and low-growth alleles represent ones contained in the high- and low-growth haplotypes, respectively. TF displayed below the grayscale bar indicate that their motifs are created by the low-growth alleles whilst upper ones exist in the case for high-growth alleles.

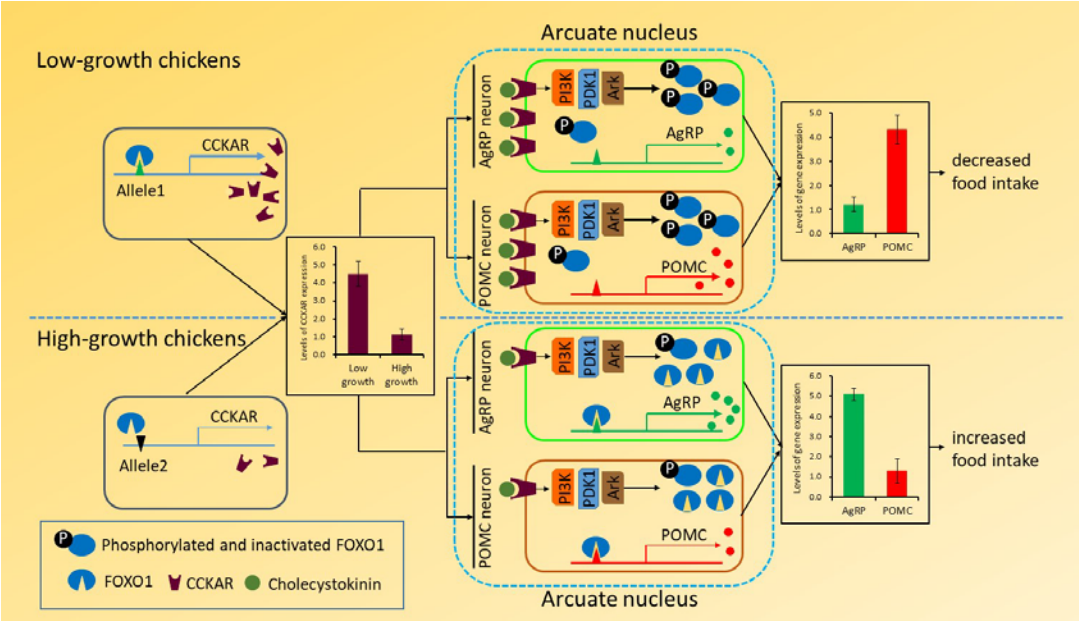

Figure S4 An interrelationship model of CCKAR and FOXO1 in appetite regulation

This interrelationship model is proposed based on regulatory roles of FOXO1 in *AgRP* and *POMC* expression and association of CCK signaling with expressions of *AgRP* and *POMC* [16,35,36]. In the interrelationship model, FOXO1 is a putative TF activating *CCKAR* expression. In appetite-regulating neurons, FOXO1 activates *AgRP* expression and suppresses *POMC* transcription, but phosphorylation of FOXO1 suppresses FOXO1-DNA interaction, which reverses expression profiles of *AgRP* and *POMC* [29]. The CCK signal is hypothesized to activate the phosphorylation of FOXO1 via the PI3K/PDK1/PKB–FoxO1 signaling axis. In high-growth chickens, decreased expression of *CCKAR* will inhibit the phosphorylation of FOXO1, which produces an orexigenic effect. In low-growth chickens, higher expression of *CCKAR* facilitates the phosphorylation of FOXO1, which inhibits appetite.
